# Report of the Second International Symposium on Molecular Epidemiology in Childhood Leukaemia and Embryonal Tumours, Rio de Janeiro, Brazil

**DOI:** 10.3332/eCMS.2008.86

**Published:** 2008-06-05

**Authors:** MS Pombo de Oliveira, S Ferman, B de Camargo

**Affiliations:** 1 Program of Pediatric Hematology-Oncology Research Centre, Instituto Nacional de Câncer, Rua André Cavalcanti 37 CEP: 20231-050, Rio de Janeiro, Brazil; 2 Oncopediatric Service-Hospital do Câncer, Rio de Janeiro, Brazil

## Abstract

The recent International Symposium on Molecular epidemiology in Embryonal Tumours and Paediatric Leukaemia was held on 4–6 March 2008 in Rio de Janeiro, Brazil. It proved a very productive meeting in which studies relating to genetics, therapeutical trials, identification of risk factors in acute leukaemia neuroblastoma and Wilms’ tumours were presented. Over 120 participants gathered for three days of fruitful discussions, including representatives of paediatrics, haematology, laboratory, epidemiology and pathology. Debates were held about strategies of applications of important biomarkers for clinical trials. Highlights of each of the scientific presentations are summarized below.

## Introduction

The First Symposium on Molecular Epidemiology in Childhood Leukaemia was held in June 2006 to gather colleagues to discuss and work together on molecular epidemiology in childhood acute leukaemia. During this meeting, the preliminary results of the Brazilian Collaborative Study Group of Infant Acute Leukaemia (BCSGIAL) were discussed, and the commitment of several institutions was consolidated. Since then a centralized biological reservoir of material in INCA has allowed studies on environmental factors and biomarkers in childhood leukaemia to start. A second symposium was organized for the results of previous studies, and to start a new programme for the study of molecular epidemiology in paediatric embryonic tumours. The content of the programme predominantly includes acute leukaemia Wilms’ tumour (WT) and neuroblastoma.

This second symposium was opened by Marisa Breitenbach, the coordinator of the Instituto Nacional do Câncer Rio de Janeiro. Paulo de Biase (Director INCA Rio de Janeiro) welcomed all the participants from abroad and from other parts of Brazil. He acknowledged the importance of international meetings for the exchange of knowledge in order to improve the understanding and treatment of childhood cancer. Marisa Breitenback emphasized the dedication of the participants of the Haematology-Oncology Paediatric Program carrying out multidisciplinary research as an exemplary approach to the challenges of cancer research. As the official spokeswoman for the institute, she discussed the development of the Instituto Nacional de Câncer in promoting the interaction between basic scientific researchers and members of the medical society, reinforcing the link between education, research and clinical innovations. Increased research funding and the establishment of advanced translational research infrastructures were also discussed.

## Acute leukaemia

The two-hit model, as a paradigm explanation for the etiopathology of childhood common acute lymphoblastic leukaemia (c-ALL), was reviewed by Joseph Wiemels (UCLA-San Francisco, USA). Sustained by the molecular epidemiological data, he demonstrated that specific gene fusions (***TEL-AML1***) occurring prenatally are important, but not exclusive markers for clinical phenotype. The biologic pathway of ***TEL-AML1*** leukaemia was further discussed and candidates for the second post-natal hit were outlined. This second hit could be related to infectious disease, pesticide exposure and the deletion of ***p*** arm of chromosome 12. The biological evidence of ***Ras*** mutations occurring post-natally was also presented (from Guthrie card data). Loss of heterozygosity in a common region of chromosome 14 was also disclosed as an important factor in the scenario of ALL pathogenesis. In summary, the two-hit model for the development of c-ALL was proposed; the challenge is now to understand the role of delayed infections, exposure to pesticides and the impact of gene promoter methylation signature in its pathogenesis.

Two studies, (1) the Northern California Childhood Leukaemia Study by Catherine Metayer (University of California, Berkeley, USA) and (2) from BCSGIAL by Sergio Koifman (Escola Nacional de Saúde Publica, Brazil), addressing parental exposure and environmental factors, were the subject of a roundtable discussion.

The epidemiological studies conducted in Berkeley focused on exposure to home and agricultural pesticides, the timing of exposure and carcinogenesis. On the basis of the two-hit model hypothesis, the design of these studies analysed the questionnaires compiled by mothers of children with leukaemia, taking into account geographic distribution and biological markers. Preliminary data demonstrated that the use of paint at home during the post-natal period presented an OR of 1.37 95%CI (1.05–1.79) for ALL in the offspring, whereas the risk of AML was OR 2.54 95%CI (1.19–5.42) for petroleum solvents. The data were analysed according to cytogenetic status. Although based on small numbers, the association found with paint and solvent exposures diverged markedly, depending on the abnormal karyotype, suggesting that a role for subtype-specific pathways and genetic susceptibility. Additionally, increased risks of childhood ALL was associated with increased dust levels of herbicide combustion sources and organochlorine compounds.

Sergio Koifman presented a hospital-based case-control study in which the participants were enrolled between 1999 and 2006 with ALL, AML and a control group. Moderate increased risk for acute leukaemia was associated with a birth weight of 3500 gm or more (OR 1.88 95% CI 1.29–2.15). Increased ORs were observed comparatively to quartile of birthweight distribution: OR = 1.46 95% CI 0.79–2.71 in the second quartile OR = 3.18 95% CI 1.83–5.53 in the third quartile and OR = 3.57 95% CI 2.07–6.18 in the fourth quartile. Similar trends were observed according to birthweight strata for ALL and AML. Some hypotheses for the biological mechanisms relating birthweight to the development of infant acute leukaemia (IAL) were proposed. One of them being hormonal intake during pregnancy, which in a previous analysis showed a high association with IAL with an OR 8.76 95% CI 2.85–26.93 (Pombo-de-Oliveira MS 2006).

Cellular and immunomolecular profiling of childhood leukaemia in Brazil was described by Maria S Pombo-de-Oliveira as a consequence of the Brazilian immunomolecular study group. The results according to immunophenotyping and genotyping profiles demonstrated the same frequencies of biomarkers as described worldwide. There was a positive change in the process of identification of childhood leukaemia and an improvement in diagnosis. The amount of biological samples sent for diagnosis increased threefold in 2000–07 (*n*=3618) compared to 1990–9 (*n*=1003). Increasing expectations of these improvements was hampered by the complexity of the overlap from local infectious diseases. Differential diagnosis of local parasitic disease is under evaluation. Kala-zar, an infectious disease caused by ***Leishmania chagasi,*** an important medical problem mainly in the Northeast and Central plateau regions (1310–7616 cases reported annually), is either treated with glucantine or amphotericin B. A particular topic under exploration is whether Kala-zar, or its treatment could represent the second hit for the development of childhood c-ALL. A proposal to start an exploratory epidemiological study combined with genomic signature was positively accepted. Other questions were raised, such as previous exposure to medications leading to bone marrow abnormalities and the standardisation of protocols to identify minimal residual disease in ALL.

An international collaboration, supported by the International Agency on Research for Cancer (IARC), was described by Catherine Metayer and Beatriz de Camargo (INCA, Brazil). Catherine Metayer reviewed the history of Childhood Leukaemia International Consortium (CLIC), established in collaboration with UC Berkeley and IARC to overcome the limitations of childhood leukaemia studies, due to limited number of samples especially for rare subtypes. The objective is to combine existing data from several comparable case-control studies conducted in Northern Europe, Southern America and Asia to increase the numbers to approximately 8000 childhood leukaemia and 13,000 controls. Because of the participation from New Zealand, Seoul, Australia, United Kingdom, Quebec, Montreal, Italy, France, Germany, Brazil and California (USA), 8106 cases and 13,303 controls have been collected since 1989. Fifty per cent of these have DNA specimens available.

Following on, Beatriz de Camargo presented the new proposal from the IARC on an International Study of non-CNS Embryonal Tumours (ISET). The rational for this proposal is the lack of large-scale aetiological studies on solid embryonal tumours. The study will be started with a pilot project on WTs and neuroblastoma. The objective of the full scale study is to understand the aetiology of embryonal tumours, focusing on genetic susceptibility prenatal factors and neonatal exposures as well as aspects of molecular epidemiology, including epigenetic profiles, DNA repair capacity and mutation patterns.

Dual role for ***NFAT*** transcription factors in regulation of apoptosis cell proliferation and transformation of T and B cells was presented by João Viola (CPq-INCA). The functional role of ***NFAT***1 and 2, demonstrated in knockout mice for the first time, indicated a dichotomous role of NFAT family members in cell cycle control. This should be evaluated in human models as NFAT1 suppresses cell transformation of the neoplastic cells may act as a tumour suppressor.

Genetic polymorphisms and susceptibility to childhood acute lymphoblastic leukaemia was discussed by Carlos Scrideli (Faculdade de Medicina de Ribeirão, Preto, São Paulo). ***CYP2D6, CYP3A5, EPHX1, MPO, NQO1, NAT2, TS, XRCC1*** and ***XPD*** polymorphisms were studied in a group of children with acute lymphoblastic leukaemia and ***CYP2D6*** (del A and G/A), ***CYP3A5*** (*3) and ***XPD*** (A/C) may have an important role in increasing the risk of ALL in children. ***EPHX1*** (T/C and A/G), ***TS*** (tandem repeat), ***NQO1*** (C/T) and ***MPO*** (G/A) polymorphisms may have a role in protection against ALL.

Juliana Godoy Assumpção discussed a study on the role of ***TP53 R337H*** mutation in paediatric ALL, which was tested in a group of 208 children treated at the Centro de Investigações Infantil D Boldrini Campinas, São Paulo, during the period 2001–07. Although this alteration is already known to be associated with adrenocortical tumours in Brazilian children, no association of the ***TP53 R337H*** mutation was found in ALL.

A large review of the literature in case-control studies in AML was carried out by Maria Lucia de Martino Lee (Instituto de Oncologia Pediatrica/UNIFESP São Paulo). Risk factors such as alcohol during pregnancy, parental exposures to pesticides and drugs exposure were mentioned. Comparison of the incidence of chromosomal abnormalities in different regions of the world was also reviewed. The proportion of promyelocytic leukaemia in Hispanic children varies with the series of cases considered, ranging from 13.0% to 37.5%. The same can be observed in the Brazilian series of AML cases. A proposal for a new multi-institutional study of chromosomal abnormalities in AML was disclosed by Maria Luiza Macedo Silva (CEMO-INCA).

The integration of flow cytometry into AML protocols, to assess levels of minimal residual disease, was presented by Mihoko Yamamoto (UNIFESP São Paulo). The detection and clinical significance of aberrant phenotypes associated with molecular detection of fusion gene was reviewed. The mutation analysis of ***FLT3*** in AML was presented by Ilana Zalcberg (CEMO-INCA). This study used an innovative approach that searches for unidentified activating ***FLT3*** mutations and ***NPM1*** abnormalities.

## Embryonal tumours

Childhood cancers originate from embryonal cells during prenatal and post-natal development. Defects in any of the pathways that control development, tissue growth and differentiation can promote transformation, making these cells particularly prone to carcinogenesis. These pathways were elegantly discussed by Rogier Versteeg (Academic Medical Centre, The Netherlands) specifically in neuroblastoma. Embryonal tumours have clinical and epidemiological common features that suggest common pathways. Features such as age of onset, birthweight and association with congenital abnormalities were reviewed by Beatriz de Camargo. It is well known that children with cancer have a high incidence of malformation syndromes. WT has been reported in association with more than 50 different clinical conditions and several abnormal constitutional karyotypes. High-risk syndromes (>20%) associated with WT include Denys-Drash syndrome, Perlman syndrome, Familial WT and WAGR syndrome. Beckwith-Wiedman syndrome and Simpson-Golabai-Behmel syndrome are associated with a moderate risk of 5–20%. Isolated hemi-hypertrophy and several other syndromes have been described in association with WT. High birthweight has been identified as a possible risk factor for WT in several population-based studies, but results are not consistent outside the context of overgrowth disorder association. Low birthweight is associated with hepatoblastoma in several reports. For rhabdomyosarcoma, the histological alveolar subtype is associated with heavier birthweight, suggesting that this should be analysed separately. Costello syndrome and type 1 neurofibromatosis is associated with embryonal rhabdomyosarcoma. All embryonal tumours are associated with Beckwith-Wiedman and some other overgrowth syndromes. Birthweight and congenital anomalies suggest that there are critical time points during the gestation and common risk factors.

An overview of the recommendations for genetic counselling in children with embryonic tumours was presented by Fernando Vargas (INCA-Brazil). Although the percentage of childhood cancers with a clearly inherited predisposition is low, sentinel karyotyping should be beneficial in children with a genetic abnormality. Structural chromosomal aberrations such as del (1p36), del (8q24.1), del (9p24), del (11p13) and del (13q14) are strongly associated with neuroblastoma, osteosarcoma, gonadoblastomam WT and retinoblastoma. The criteria for genetic testing and genetic counselling were presented.

Down’s syndrome (DS) offers a natural genetic model for cancer protection. A review of the association of DS with paediatric neoplasm was presented by Isis Magalhães (Hospital de Base Brasilia). A study of 17,897 Americans with DS confirmed the higher incidence of leukaemia and testicular cancer and a lower incidence of breast cancer. A study of 3581 persons with DS in Finland has also reported a higher risk of leukaemia and testicular cancer, and a lower risk for solid tumours than in the general population. Aneuploidy of chromosome 21 has been linked to leukemogenesis, as trisomy or tetrasomy of chromosome 21 is common in childhood ALL. In recent years, it has been reported that somatic mutations in the X-linked ***GATA1*** gene are present in AML blasts of DS children. In order to test whether specificity of ***GATA1*** mutations would be a helpful tool to distinguish different haematopoietic disorders in children with DS, samples from a cohort of DS children were analysed. ***GATA1*** mutations in haematological clonal disorders were found in 75% of AMLs. Although inherent mutation leading to the production of the short isoform of ***GATA1*** is associated with impaired megakaryopoiesis and erythropoises, it does not lead to leukaemia in the absence of trisomy 21. Therefore, trisomy is essential for the leukemogenesis with additional changes characterized by a third hit.

The correlation between histology and molecular features was reviewed by Paulo Faria (INCA-Brazil). Chromosomal abnormalities including reciprocal translocations, deletions, mutations and amplifications have a specific histopathology type. ***WT1*** mutations are associated with intralobar nephrogenic rests and rhabdomyomatous differentiation. Gene fusions such as ***PAX3-FKHR and PAX-7-FKHR*** are associated with alveolar rhabdomyosarcoma, while other abnormalities are present in embryonal rhabdomyosarcomas. The Ewing’s sarcoma family of tumours has several histology features, but all share a common molecular pathogenesis (chromosomal translocations of EWS gene and one of the several members of ETS family of transcription factors). Recognition of morphological and molecular features is important for determining biological behaviour. Classifications of paediatric neoplasms should include morphology immunohistochemical and molecular biologic aspects. The importance of the cancer biobanking was highlighted.

As embryonal tumours differ from adult tumours, it is important to study them under the perspective of organ development, taking into consideration histological differentiation. WT morphology is similar to specific development phases of kidney morphogenesis.

In a study, Dirce Carraro (Hospital AC Camargo, São Paulo) demonstrated that the blastema component preferentially presented gene expression patterns similar to the earliest stage of kidney development, suggesting that morphological aspects acquired by WT are dependent on the temporal and spatial occurrence of the mutation in embryonic blastema during kidney development.

The evolving story of WT biology was reviewed by Jeff Dome (Children’s National Medical Center, Washington, DC, USA). WT, whose pathogenesis was once thought to follow the same single-gene, two-hit model as retinoblastoma, has instead become a paradigm for genetic heterogeneity and complexity. Several genes have been implicated in its development, most of which are associated with familial disease or malformation syndromes that affect only a small fraction of cases. Constitutional deletions of ***WT1*** at 11p13 is well established as the genetic basis for WAGR (WT aniridia genitourinary anomaly mental retardation), while germ line mis-sense mutations in ***WT1*** are responsible for WT occurring as part of Denys-Drash syndrome. Several candidate genes at the second WT locus ***WT2*** have been described. ***WT1*** is altered in only approximately 20% of WT. Beta-catenin mutations have been described as present in 15%, and Wnt signalling pathway is probably implicated in this neoplasm. Recently, a novel gene ***WTX*** at Xq11.1 was reported to be mutated in WT. Genetic abnormalities are known in about 75% of tumours: 15% ***WT;*** 30% ***WT2;*** 30% ***WTX***. Much progress has been made in understanding the biology of WT.

An 11-year study carried out in Southern Brazil (Mara Pianoviski, Curitiba, Brazil) emphasized that there was a significant delay in diagnosis, resulting in less successful treatment and poorer survival in children less than two years of age. The frequency of amplified ***N-myc*** was higher than that reported by other studies. In a series of patients from Parana, Southern Brazil, all the male patients with amplified ***N-myc*** and unfavourable histology who died were of an older age and a more advanced stage of disease, which suggested late diagnosis.

A study carried out in INCA on the amplification of ***N-myc*** in tumours was reported by Arissa Ikeda (INCA, Brazil). Twenty-nine tumour samples, 14 fresh and 21 paraffin embedded were analysed. Nine had amplifications and this was correlated with the outcome. ***N-myc*** evaluation is routinely recommended to all patients for risk classification and appropriate treatment strategy. However, cost–benefit effectiveness in our country was discussed, and this type of evaluation was recommended mainly in children with neuroblastoma, with localized disease stage INSS 2A/2B/3, stage 4S and INSS stage 4, at less than one year old .

A presentation of treatment strategies was given by Jeff Dome for WT and Barbara Hero (University Hospital Cologne Germany) for neuroblastoma. There are two major approaches in WT, one with pre-chemotherapy and the other with surgery first. Both approaches have similar overall survival. Modern approaches to WT treatment include both reduction of therapy and augmentation of therapy. Reduction strategies are being tested by SIOP (Europe and Brazil) and COG (USA), and these propose the omission of doxorubicin in stage II/III, omission of chemotherapy in stage I small tumours and omission of lung irradiation for rapid responders. Augmentation therapies are being tested in patients with tumours presenting LOH 1p/16q, patients with tumours histology after chemotherapy blastema-predominant stage IV, patients with anaplasia. New agents are becoming available; the most promising are the microtubule inhibitors ixabepilone and ABT-751. An international relapse protocol will be started with ICE (ifosfamide carboplatin vincristine)-topotecan as an induction phase, followed by randomization to BMT (autologous bone marrow transplant) or maintenance. The treatment approach for neuroblastoma goes from minimal intervention in stages 1, 2, 4S and stage 3 less than two years, to maximal treatment in stage 4 patients over one year old and tumours with ***n-MYC*** amplification. A wait-and-see strategy in localized disease and children less than one year old is recommended. Induction surgery, megatherapy, radiotherapy and consolidation therapy are given to high-risk patients. In conclusion, risk adapted treatment is necessary for children with neuroblastoma. Data from an experience with a dendritic-cell tumour, in which cell hybrid vaccination was given every six weeks in advanced relapse neuroblastoma (Alexandre Barbuto, ICB USP São Paulo, Brazil) indicated that immune function, recovery and time to tumours progression was delayed.

## Prognostic risk classifications in ALL Wilms’ tumour and neuroblastoma

The survival rate of childhood cancer has exceeded 80% and classification of prognostic risk is essential for treatment strategies. Current prognostic factors for WT neuroblastoma and acute lymphoblastic leukaemia were reviewed by Jeff Dome, Barbara Hero, Silvia R Brandalise, (Centro de Investigações Infantil D Boldrini Campinas, São Paulo) and Marco Borato Vianna (Universidade Federal de Minas Gerais, Brazil), respectively.

In WT, the most important negative prognostic factor is histological findings of anaplasia. Recent research has focused on identification of possible molecular genetic markers. In the study of NWTS-3–4, loss of heterozygosity for markers on chromosome 16q and 1p was associated with poorer two-year relapse-free and overall survival rates. This observation was tested on NWTS-5 and confirmed in tumours with loss of heterozygosity jointly for both chromosomes 1p and 16q. The level of telomerase expression in tumours has been correlated with risk of tumour recurrence. Gain of chromosome 1q is associated with loss of 1p and 16q and is under evaluation as a risk factor. Gene expression arrays were of limited value until now. Tumours are heterogeneous with regard to their relapse signatures.

The major prognostic variables in neuroblastoma are age at diagnosis stage, ***N*-*myc*** amplification status, tumours cell ploidy, 1p deletions and tumours pathology. The most important independent factor is still ***N-myc*** amplification.

Prognostic values in ALL are age, white blood count at diagnosis, early response to treatment, cytogenetics, immunophenotyping, molecular biology and *in vitro* drug sensitivity. This knowledge is useful for stratifying patients into low, moderate and high-risk groups.

The Brazilian Cooperative Study Groups (GBTLI) were reviewed by Silvia R Brandalise. The GBTLI-93 stratified children with ALL into three groups: very low risk (age between >=1 and <= 10 years, white blood count at diagnosis <=10000/mm, no mediastinal mass without CNS involvement), low risk (age between >=1 and <= 10 years, white blood count at diagnosis between 10.000/mm and < 50000/mm with or without mediastinal mass) and high risk (ageƏ year or >=10 years, white blood count at diagnosis >=50000 with or without CNS involvement). Overall survival was 84%, 81% and 57%, respectively. In a following study, GBTLI-99 patients were classified as high risk (age Ə year or > 9 years white blood count < 50000) and low risk (age between one and 10 years white blood count < 50000). Although immunomolecular results were not considered for risk definition, immuno-phenotyping, DNA index and molecular biology were performed in 92%, 64% and 51% of the cases, respectively. Event-free survival was 59% for high-risk and 78% for low-risk patients. Socio-economic factors were discussed by Marco Borato Vianna. Overall survival for children with ALL living in underdeveloped or developing countries is clearly inferior to those in developed countries. Environmental factors such as nutritional status, total income of the family, height for age and ethnicity have been published as prognostic factors. Compliance, treatment, intensity and genetic polymorphisms are also important in predicting outcome. Additional efforts should be undertaken to clarify mechanisms of socioeconomic influence that are clearly multifactorial.

The visibility of the Brazilian network, so-called ***Rede de Atenção Oncológica,*** has been improving the research and outcome in paediatric oncology (presented by Marco Porto, INCA, Brazil). The aim of this enterprise is mainly to reduce mortality and improve quality of life of cancer patients in Brazil. There is a strong commitment and participation from government and non-government organisations. The epidemiological and immunomolecular study (sponsored by INCA FAF/Swiss Bridge Foundation) is currently working very well. Cases are notified, samples are sent by courier overnight to be analysed and their results promptly available online. The establishment of this network represents a potential contribution to assist the demands of clinicians and researchers.

## Conclusions

Discussion was stimulated by the organizing committee (Maria S Pombo-de-Oliveira, Sima Ferman and Beatriz de Camargo) with colleagues from all over Brazil having the opportunity to exchange experiences. The meeting provided an outstanding overview on molecular epidemiology in paediatric leukaemia and embryonal tumours. International scientific collaboration provided direction for future research, which has been, or will be, implemented.

